# The Recent Press Excursion over the E. T., V. & Ga. R. R.

**Published:** 1884-09

**Authors:** 


### THE RECENT PRESS EXCURSION OVER THE EAST TENNESSEE, VIRGINIA AND GEORGIA RAILROAD.

### THROUGH THE SHENANDOAH VALLEY TO HAGERSTOWN—ON TO THE CITY OF BALTIMORE—THENCE TO GETTYSBURG AND CARLISLE, AND HOME AGAIN.

The recent trip of the Georgia Press Association, by invitation of the East Tennessee, Virginia and Georgia Railroad Company, and under the care of its specially designated agent, constituted an episode which, to us, was full of enjoyment. Nothing is so refreshing and so rejuvenating as travel by comfortable conveyance, in pleasing companionship, into new and charming regions, and the good effects seem to be increased when the opportunity for such pleasure comes in the nature of a generous offering.

We would be strangely unappreciative were we to pass over this *editors' jaunt* without special notice. Indeed, we think the company that extended the invitation, and so liberally and hospitably dispensed its attentions *en route*, deserves our sincere gratitude.

Boarding the Pullman Palace Sleepers of the East Tennessee, Virginia and Georgia Railroad at its depot in this city on the night of the 23d ult., we were soon out of sight and sound of Atlanta, speeding, with merry hearts, northward over this magnificent road. There were in our party about seventy persons, fifty males and twenty females, and not one of the number seemed to experience a single lingering care to disturb anticipation. Even the matrons who had left the usual number of little responsibilities at home appeared, for the period, to have consigned them to forgetfulness and the care of such as would guard them well until this season of pleasure was past.

Our company comprised, in the main, the leaders of progress, and were of all ages, from those just beginning to grapple with the stern realities of business life to the veterans who had labored long and well for the good of their country and their race. On this occasion, however, the distinctions of age were lost and all were young and happy.

Breakfasting at Cleveland, a flourishing town of lower East Tennessee, we again boarded the train and sped on through that beautiful and productive region, each inspired by the continued loveliness of the landscape that feasts the eye on this line of travel.

By noon we were at Rogersville Junction, where our party did full justice to a splendid dinner, dispensed, as our young friend of the *Constitution*—Mr. Clark Howell—has announced, by "ten buxom lasses lithesome of form and rosy cheeks, buoyant and beautiful." By the way, it was difficult to determine which he enjoyed the more, the repast or the fair attendants. We are indebted to this talented and companionable young gentleman for much of the rarest pleasure of this pleasurable tour. Young, full of vivacity, a close observer of persons and things, brilliant, cultivated and intelligent, he is indeed "a worthy son of his distinguished sire."

In due time we reached the town of Bristol, situated on the line which divides Tennessee from the "mother of States and of statesmen." As we passed over into the latter a thousand proud memories of her history crowded upon us, and we felt that we were on little else than holy ground.

At this place the line of the E. T., Va. and Ga. R. R. terminates, so we boarded the cars of the Bristol and Roanoke division of the Norfolk and Western Railroad and passed on to the town of Roanoke, where we connected with the Shenandoah Valley Railroad.

This line traverses a country unsurpassed for beauty of scenery or fertility of soil. We have neither time nor space in which to give a faithful description of this valley over which the everlasting hills stand sentry.

Pursuing this route we passed very near to the Natural Bridge and to the Cave of Luray, both objects of interest to the tourist, and near its northern terminus we crossed the battle-field of Sharpsburg, made memorable by the conflict of armies and the signal victories won by those who wore the gray, and who fought, against heavy odds, for a cause which is none the less sacred because it is "LOST." As we passed over this field, hallowed by the sacrifice of heroes, a feeling of profound sadness came upon us, intensified as the names of our gallant leaders were mentioned who have "crossed over the river and rest under the shade of the trees."

At Hagerstown, the northern terminus of the Shenandoah line, we were transferred to the Western Maryland R. R, and set out for the city of Baltimore under special conduct of President Hood of this road and in company with several other distinguished railroad officials. Passing over the Blue Ridge, a panorama is unfolded which man cannot describe. The scene is a happy blending of the beautiful and the sublime, the mountains on the one side and the valley of the Cumberland on the other contributing to this result.

In due time we arrived at Baltimore, where we were met and received with genuine hospitality by a delegation of the press of that city, and conducted to that elegant house known as the Carrollton. There everything that a refined taste or a generous hospitality could devise was ours.

On Friday, as the guests of the Press Association, the Chemical and Fertilizer Exchange, and the Merchants' and Manufacturers' Association of Baltimore, we sailed out on the Bay on the steamer Westmoreland. We were accompanied by the Mayor and quite a number of other distinguished citizens and their wives. This was indeed a field day, and the ovation extended us manifested its most enjoyable features. Speeches of welcome by Mayor Latrobe and others, and responses from the good talkers of our own party, made us all feel that there was a brotherhood in operation which was replete with friendship and kindly sympathy.

On Monday the main body of our company took leave of this patriotic and hospitable seat of commerce and made our way over the H. J. H. & G. R. R. to Gettysburg, a place ever memorable as the battle-ground on which the heart of the Southern cause was fatally pierced. En route to this historic point, our train was boarded by Col. Robert H. Thomas, of Mechanicstown, Secretary of the Pennsylvania Press Association, and a number of distinguished gentlemen and handsome ladies. They came to show us the hospitalities of their grand old State, and this they did most royally. Secretary Thomas is an elegant and genial gentleman, whose kind attentions will not be forgotten. He neglected nothing which could contribute to the highest enjoyment of his guests, and wrote his name on every heart of the tourists by his faultless and unremitting care for our comfort and pleasure.

Arriving on the battle-field, we visited, under the guidance of our Pennsylvania friends, the various localities of special interest in connection with the great conflict, and saw in fancy the sublime scene of army grappling with army in terrific strife and with dauntless courage, the one fighting for the right of self-government, the other to preserve the Union of our fathers. Both were actuated by their convictions of duty, and they fought as armies had seldom fought before. Every sound was merged in the rattle of musketry and the incessant roar of artillery, and as the determined combatants rushed to engage in a closer struggle, the very air was filled with the missiles of death, making a sacrifice of life and blood—the life and blood of patriots and heroes. But 'tis all over now; the issue of the combat was against the Southern army; the Union was preserved, and we rejoice to know that "the bloody shirt" which was so long flaunted in our faces by the *ungenerous* factions of the North has been buried by the *noble* and *patriotic* citizens of that wonderfully-developed and progressive section.

We talked over, as reconciled brethren, the events of that memorable period, and all joined in common rejoicings that the asperities and prejudices immediately succeeding that contest are passing so rapidly and thoroughly away.

One of our party, Mr. Penn, of Monticello, was a participant in the terrible battle of Gettysburg, and was there severely wounded. He lived over again, in imagination, the perils of that occasion, and with a degree of enthusiasm quite natural, recounted his experiences, pointed out the positions of the integral part of the southern army, and, we doubt not, experienced the while a silent but profound feeling of gratitude for that providence which preserved his life.

From Gettysburg we went to the Indian school at Carlisle, and were much edified by what we saw. About two hundred and fifty Indian youths, fresh from their native wilds, are being educated at this school. Of these about one hundred are females. They appeared well satisfied and to be making good progress in their studies. These pupils will, when their education is complete, go back to their several tribes, and are expected to prove civilizing agents of their people.

Our party, satisfied with enjoyment, and remembering the obligations of duty at home, turned from this interesting locality Southward—stopping on the way at Pen-mar, Blue Mountain, Luray Cave and Natural Bridge. At each place we were treated with that same generous hospitality that marked our stay at other places.

During our entire journey to Baltimore we were accompanied by Mr. Samuel H. Hardwick, the special agent of the East Tennessee, Virginia and Georgia Railroad Company, to whom, for unremitting attentions and a thousand kindnesses, our party will be ever grateful. His representation of the great Company to whom we were indebted for this tour of pleasure, was most honorable and efficient. This fact has been recognized by appropriate resolutions and a memento which we hope he will cherish as the gift of a grateful friendship.

We are all at home again, refreshed and happy, our tour not having been disturbed by a single accident, and we offer our profound respects and gratitude to the E. T., Va. & Ga. R. R. Company.

